# Cohort Profile Update: The British Regional Heart Study 1978–2018: 40 years of follow-up of older British men

**DOI:** 10.1093/ije/dyac122

**Published:** 2022-06-03

**Authors:** Rachel Kimble, Gillian McLellan, Lucy T Lennon, A Olia Papacosta, John C Mathers, S Goya Wannamethee, Peter H Whincup, Shenna E Ramsay

**Affiliations:** Population Health Sciences Institute, Newcastle University, Newcastle upon Tyne, UK; Population Health Sciences Institute, Newcastle University, Newcastle upon Tyne, UK; Department of Primary Care and Population Health, University College London, London, UK; Department of Primary Care and Population Health, University College London, London, UK; Population Health Sciences Institute, Newcastle University, Newcastle upon Tyne, UK; Department of Primary Care and Population Health, University College London, London, UK; Population Health Research Institute, St George's, University of London, London, UK; Population Health Sciences Institute, Newcastle University, Newcastle upon Tyne, UK

Key Features

- The British Regional Heart Study (BRHS) consists of an all-male cohort of 7735 participants aged 40–59 years, first recruited in 1978–80 and followed up over 40 years to 2018.
- The original focus of the BRHS was to investigate regional variations in cardiovascular disease (CVD) mortality across Britain, but the objectives widened to examine other key health outcomes related to CVD and associated with healthy ageing, such as physical function, disability, frailty, dental health, malnutrition and sarcopenia.
- The latest physical examination (40-year follow-up) of the cohort, conducted in 2018, hosted 667 men and another 1009 responded to postal questionnaires, all aged 78–99 years. This follow-up enhances prospective data at very advanced ages by building on the previous examination in 2010–12 at 71–92 years.
- The new measures conducted within this cohort’s 40-year follow-up include extended objective and subjective oral health measures, subjective appetite and malnutrition measures, and anthropometric measures associated with lower body extremity muscle mass.
- The BRHS has maintained existing collaborations and remains open to new collaborative projects. Please contact Lucy Lennon [l.lennon@ucl.ac.uk] for more details.

## The original cohort

**Figure 1:**
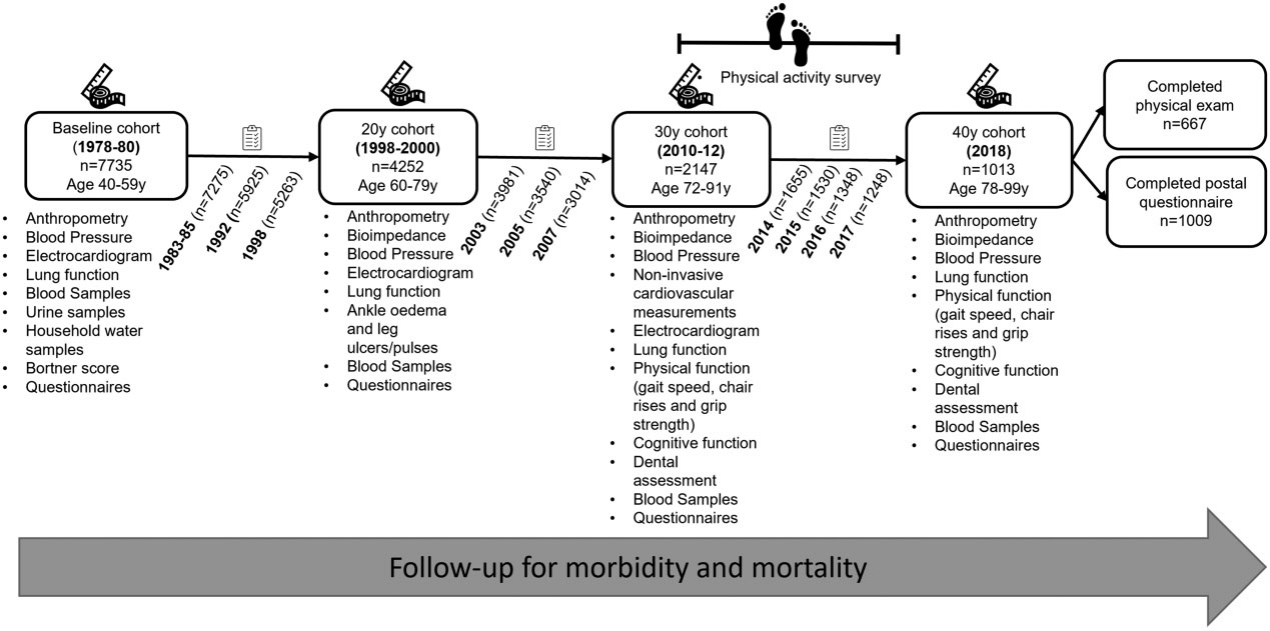
British Regional Heart Study (BRHS) follow-up time line, including attrition and mortality since recruitment in 1978–80

The British Regional Heart Study (BRHS) cohort is a socially and geographically representative population-based sample comprising men (initially aged 40–59 years) recruited from a single general practice in each of the 24 British towns representing all major British regions in 1978–80.^1^ The initial aim of the study was to investigate the occurrence and determinants of both individual and geographical variation in cardiovascular disease (CVD). At the 30-year follow-up, when the cohort were aged 71–92 years (2010–12), the data collection addressed a wider range of disease, health conditions and determinants relevant to CVD and healthy ageing. Cohort data were linked to area-level deprivation (‘index of multiple deprivation’ for England, Wales and Scotland) and hospital data [Hospital Episode Statistics (HES)]. Other measures added to the study at that time included, but were not limited to, dental health, physical function, physical activity and cells for DNA sequencing, as well as survey data relating to cognition, overall health, and behavioural and psychosocial factors, as previously described.^2^Figure 1 illustrates the detailed objective and subjective markers collected from baseline (1975–78) to the 40-year follow-up (in 2018). Physical examinations of the cohort have been conducted at baseline and thereafter at 20 years (1998–2000), 30 years (2010–12) and 40 years since baseline (2018). The cohort has been followed up annually since 2014 by postal questionnaires.

## What is the reason for the new data collection?

With increased life expectancy, population ageing and increased prevalence of long-term health conditions, disability and multimorbidity, there is an urgent need for robust, longitudinal evidence to help understand the risk trajectory of age-related diseases and the behavioural and biological determinants of healthy ageing. Moreover, reduced cognitive function, lack of independence, admissions to institutional care and hospitalization are the greatest concerns of older populations and pose inherent economic, personal and societal costs.^3^^,^^4^ Consequently, understanding factors related to these outcomes in later life would not only have economic benefits but would also have the potential to inform policy makers and improve the health, wellbeing and quality of life of older adults. Based on this rationale, the BRHS cohort participants were invited for a further follow-up ∼40 years after their original recruitment into the study. The cohort participants completed a detailed postal questionnaire (July–December 2018) and a physical examination (August 2018–January 2019) when aged 78–98 years. At the beginning of the study (1978–80), 7270 (94%) men were employed (including being in the armed forces); whereas in 2018, of the remaining participants 3.8% (vs 5.8% at 2010–12) reported spending any time in paid work. Transitioning from work to retirement or unemployment in later life presents major challenges including, but not limited to, financial insecurity^5^^,^^6^ and social isolation,^7^ which increase in prevalence with age. Unlike other studies of older old adults,^8^ the BRHS has distinctively captured this period of the adult life course from middle age into very old ages, with detailed information related to social, behavioural and biological factors in community-dwelling men.^9^ Thus, the Study has evolved accordingly to focus on issues that are more relevant to older adults. Furthermore, the longitudinal collection of objective and subjective measures since 1978–80 represents a rich dataset that is available for research to identify risk factors for chronic diseases and mortality and to explore behavioural and social factors related to healthy ageing among the surviving cohort (*n* = 1633; Figure 1). For participants who are absent from the examination and questionnaire surveys, health information is available from GP records and mortality.

## What are the new areas of research?

Ageing presents different nutritional demands associated with age-related physical and psychological dysfunction, changes in lifestyle, and the use of medication to treat chronic disease. In addition, oral health problems such as periodontal disease, xerostomia (dry mouth), tooth loss and oral-health-related quality of life problems (e.g. difficulty chewing and swallowing) impact on nutritional intake and influence the onset of malnutrition.^10^ Inadequate nutritional intake can result in a decrease in serum albumin and muscle protein wasting, and inflammation can also affect these nutritional measures.^11^ Moreover, inflammation and inadequate nutritional intake may be consequences of poor oral health, since periodontal disease is associated with increased systemic inflammatory markers^12^ and reduced diet quality.^13^ Inflammation may be a common underpinning mechanism associated with poor oral health, frailty, sarcopenia and CVD, which may be exacerbated by behavioural factors including low physical activity and poor nutrition.^14^

Physical frailty—a state of increased vulnerability to physiological and psychological stressors that diminish independence^15^—has been established as an important geriatric syndrome and is associated with cardiovascular risk factors^16^ and, more recently, with risk for mortality and the development of major adverse cardiovascular events.^17^ Frailty may co-exist with inadequate nutritional intake (malnutrition), anorexia and sarcopenia with uncertainty about causal pathways. For example, unintentional weight loss can be either a cause or consequence of chronic diseases and loss of independence in older adults. The detailed measures collected longitudinally in the BRHS will allow the prospective study of risk factors and will help in establishing the relationship between oral health, behaviours and age-related health outcomes in older adults. Collection of blood samples provides opportunities for further exploration of biomarkers and genetic phenotyping, to investigate the aetiology and preventative risk factors for frailty and other forms of morbidity related to cardiovascular ageing.

## Who is in the cohort?

At baseline, 7735 men attended a physical examination then aged 40–59 years. At the 40-year follow-up in 2018, 1633 surviving men were invited to the physical examination, of whom 667 attended (41% response rate). A postal questionnaire was sent with the invitation to the physical examination. This questionnaire was completed by 1009 (62% response rate), although four men who attended the physical examination did not complete the questionnaire, meaning there were data available for 1013 men who either completed the questionnaire or attended the physical examination. These response rates had a lower proportion attending the examination than completing the questionnaire, similar to the previous follow-ups.^2^ Key demographics of the cohort at the examination phases are provided in Table 1. The cohort is now characterized by a large proportion of oldest-old (85 years or older = 39%) community-dwelling men. As expected, those in the remaining cohort are mainly living in their own home, have higher education and are from a non-manual social class.^18^ Despite a lower proportion of manual class in the 40-year follow-up, the BRHS cohort has retained a proportion of participants from manual social class which is nationally representative of overall UK males (37%; https://www.ons.gov.uk/) and is comparable to other cohorts of older individuals in England/UK (e.g. Health and Employment after Fifty).^19^ Moreover, Table 1 shows that the spread of the cohort across Britain has remained similar since baseline over the 40-year follow-up, suggesting that the cohort contains a stable representative sample of older British men from different UK regions. This is in contrast to other studies that are either not as long in duration, not age-specific, include relatively few very old people or are based in a single geographical region.

**Table 1:** Key demographics of the British Regional Heart Study cohort at each physical examination (from those who completed questionnaires) from baseline to 40-year follow-up in 2018

Characteristic	Baseline	20-year follow-up	30-year follow-up	40-year follow-up
	(1978–80)	(1998–2000)	(2010–12)	(2018)
*N*	7735	4252	2137	1009
Age (years), median (IQR)	50.3 (10.0)	68.3 (9.0)	77.7 (7.3)	83.8 (5.2)
Social class				
Manual, *n* (%)	4428 (57.3)	2166 (50.9)	997 (46.7)	411 (40.7)
Non-manual, *n* (%)	3061 (39.6)	1966 (46.2)	1077 (50.4)	570 (56.5)
Armed forces, *n* (%)	246 (3.2)	120 (2.8)	63 (3.0)	28 (2.8)
Marital status				
Married, *n* (%)	6985 (90.3)	3467 (81.5)	1578 (73.8)	695 (68.9)
Widowed, *n* (%)	98 (1.3)	316 (7.4)	382 (17.9)	228 (22.6)
Divorced or separated, *n* (%)	−^a^	179 (4.2)	75 (3.5)	31 (3.1)
Single, *n* (%)	374 (4.8)	149 (3.5)	68 (3.2)	41 (4.1)
Other, *n* (%)	278 (3.6)	8 (0.2)	9 (0.4)	6 (0.6)
Missing data, *n* (%)	0	133 (3.1)	25 (1.2)	8 (1.2)
Living situation				
Own house, *n* (%)	–	3605 (84.8)	1880 (88.0)	935 (92.7)
Other, *n* (%)	–	531 (12.5)	227 (10.6)	61 (6.1)
Missing data, *n* (%)	7735 (100)	116 (2.7)	30 (1.4)	13 (1.3)
Geographical region^b^				
Scotland, *n* (%)	962 (12.4)	448 (10.5%)	182 (10.6%)	73 (10.9%)
North, *n* (%)	3285 (42.5)	1733 (40.7%)	661 (38.3%)	270 (40.5%)
Midlands, *n* (%)	1208 (15.6)	667 (15.7%)	267 (15.6%)	84 (12.6%)
South, *n* (%)	2280 (29.5)	1404 (33.0%)	612 (35.5%)	240 (36.0%)

Scotland: Falkirk, Ayr, Dunfermline; North: Harrogate, Southport, Burnley, Dewsbury, Darlington, Carlisle, Grimsby, Wigan, Scunthorpe, Hartlepool; Midlands: Shrewsbury, Mansfield, Merthyr Tydfil, Newcastle-under-Lyme; South: Lowestoft, Guildford, Exeter, Ipswich, Gloucester, Maidstone, Bedford.

a Not included in questionnaire.

b Regions based on those who attended the physical examination (*n* = 7735, 4252, 1722, 667, respectively).

## What has been measured?

Extensive assessments were conducted both through physical examination and questionnaires focusing on extending the longitudinal follow-up of the surviving cohort (Figure 1). In addition, new assessments (i.e. oral health, malnutrition, sarcopenia and frailty) have been added to the study to address current and emerging policy, social and health issues for older people. Table 2 provides an overview of new additional measures, including survey questions, which have been collected since the previous update (see Lennon *et al*. 2015).^2^ Furthermore, postal questionnaires and follow-up for morbidity are ongoing.

**Table 2:** New variables added to data collection in the British Regional Heart Study since the previous profile update^2^ at the 40-year re-examination (2018; cohort aged 78–99 years)

Data collected	2010–12 exam	2018 exam
Anthropometry		
Body composition (Tanita BC418 Body Composition Analyser)	✓	✓
Mid-arm circumference	✓	✓
*Calf Circumference*		✓
Physical function	✓	✓
Grip strength	✓	✓
3-min walking speed	✓	✓
5 serial chair rises	✓	✓
Non-invasive cardiovascular measurements	✓	
Dental assessments	✓	✓
Number of teeth	✓	✓
Periodontal pocket depth	✓^a^	✓
Loss of attachment	✓^a^	✓
Gingival bleeding	✓^a^	✓
*Clinical oral dryness score*		✓
*Oral lesions*		✓
*Functional pairs*		✓
*Dentures*		✓
Blood samples	✓	✓
Haematology and metabolic measures (e.g. blood counts, insulin)	✓	✓
Inflammatory and haemostatic biomarkers (e.g. CRP, IL-6, vWF)	✓	✓
Cardiac biomarkers (e.g. NT-ProBNP, troponin-T)	✓	✓
Samples stored for further biomarker and genetic phenotyping	✓	✓
Questionnaire	✓	✓
Hearing	✓	✓
Eyesight	✓	✓
Sleep patterns	✓	✓
Activities of daily living	✓	✓
*Fractures and fall injuries*		✓
*Health services (cardiac rehabilitation, hearing tests, dentists)*		✓
*Stress and illness in past 3 months*		✓
*Bladder control*		✓
Dental health	✓	✓
*Molars and chewing difficulty*		✓
*Oral hygiene*		✓
Memory	✓	✓
*Forgetfulness*		✓
Depression	✓	✓
Local environment	✓	✓
Medications	✓	✓
Personal circumstances (marital status, accommodation, feelings)	✓	✓
Diet	✓	✓
*Appetite, food and drink intake*		✓
*Taste and smell*		✓
Test your memory self-completed questionnaire	✓	✓

New additional measures available at 40-year follow-up in italics.

IQR, interquartile range.

a Six index teeth only, one in each sextant.

### Questionnaires

Since the previous update, postal questionnaires have been completed annually (2015–18), assessing factors in five broad domains: (i) overall health, diagnoses and self-reported symptoms; (ii) socioeconomic factors; (iii) health behaviours; (iv) anthropometry and nutrition; and (v) physical function in line with previous questionnaires. New measurements added to the 2018 questionnaire include the Simplified Nutritional Appetite Questionnaire (SNAQ)^20^ and the Mini Nutritional Assessment,^21^ to assess appetite and malnutrition, in addition to World Health Organization food frequency questionnaires also collected at earlier phases.^22^^,^^23^ In addition, the collection of questionnaire and physical examination data provides a unique dataset which has allowed the validation of various subjective questionnaires with objective markers (e.g. physical activity^24^ and function^25^) within the cohort, which are continued in ongoing follow-up of the cohort. Last, where possible, data collected through questionnaires are based on validated tools used in other national and international studies of ageing (e.g. the English Longitudinal Study of Ageing, the Irish Longitudinal Study on Ageing and the US Health and Retirement Survey) to facilitate pooling of data and cross-country comparisons, which will strengthen any findings of future research.

### Physical examination

In 2018, similar to the previous examination, clinical measurements were carried out by trained research nurses, with the same assessments (anthropometry, lung function, blood pressure, body composition, physical functioning and oral health) followed up into older ages. Accelerometer data (Actigraph GT3X Monitor) were collected in a subset of men included in the physical activity survey between 2010 and 2017. Additionally, the 40-year follow-up included additional measurements e.g. calf circumference. Calf circumference has been identified as an important non-invasive, cost-effective marker of sarcopenia and malnutrition.^29^ Moreover, maintaining lower extremity muscle mass is significant to mobility and disability in older adults, making it a useful assessment in key themes identified for the new follow-up.^26^^,^^27^ Additionally, oral health measurements were conducted by a dental hygienist. The new measures now include a full periodontal examination comparable to the Adult Dental Health Survey, the Health, Aging and Body composition study and the National Health and Nutrition Examination Survey. An objective marker of clinical oral dryness score^28^ was assessed at the examination, in light of recent associations in the BRHS between self-reported xerostomia and functional decline with age.^29^^,^^30^ Furthermore oral lesions, specifically those related to denture use, were recorded since a high proportion of participants now use dentures (Table 3). Finally blood samples were taken, which have been analysed for lipids and carbohydrate metabolism, and serum and plasma are stored for further analysis. Blood samples have been used to measure inflammatory and haemostatic markers (e.g. C-reactive protein, interleukins, von Willebrand factor), and cardiac biomarkers (e.g. NT-ProBNP, troponin-T). These blood samples and measures offer opportunities for further genotyping and investigation of the role of inflammatory and other biomarkers (e.g. such as C-reactive protein, interleukins, von Willebrand factor) in cardiovascular ageing and related health outcomes.

**Table 3:** Summary of characteristics of the British Regional Heart Study cohort in 2018 (aged 78–99 years) based on men who answered questionnaires (*n* = 1009) and attended physical examination (*n* = 667); values are from those whose data were available for that variable

Variable	BRHS men in 2018
Health and self-reported symptoms	
Systolic blood pressure (mmHg), mean ± SD	149.9 ± 19.4
Diastolic blood pressure (mmHg), mean ± SD	74.5 ± 11.8
FEV_1_ (L/s), mean ± SD	2.3 ± 0.6
Fair/poor self-rated health, *n* (%)	330 (33.1)
History of cardiovascular disease, *n* (%)	293 (29.4)
History of diabetes, *n* (%)	190 (18.9)
History of respiratory disease, *n* (%)	502 (51.5)
History of neurodegenerative disease, *n* (%)	41 (4.3)
Multimorbidity (≥2 of the above), *n* (%)	260 (25.8)
Polypharmacy (>3 prescribed medications), *n* (%)	601 (59.6)
Urinary incontinence in the past 12 months, *n* (%)	398 (40.6)
Socioeconomic and behavioural factors	
Current smokers, *n* (%)	20 (2.0)
Moderate to heavy drinkers, *n* (%)	27 (2.7)
Anxiety or depression, *n* (%)	202 (20.5)
Financial difficulties in past 2 years, *n* (%)	16 (1.6)
Low social engagement,^a^*n* (%)	184 (18.3)
Loneliness,^b^*n* (%)	112 (11.7)
Low physical activity,^c^*n* (%)	361 (35.8)
Fair to poor sleep quality,^d^*n* (%)	397 (40.0)
Oral health	
Edentulism (no teeth), *n* (%)	161 (16.8)
Wear dentures, *n* (%)	542 (55.4)
Less than 5 functional teeth units, *n* (%)	331 (50.0)
Periodontal disease^e^, *n* (%)	56 (9.9)
Clinical oral dryness symptom (≥1), *n* (%)	125 (18.8)
Dental lesion (≥1), *n* (%)	60 (9.0)
Anthropometry and nutrition	
BMI (kg/m^2^), mean ± SD	26.6 ± 4.0
Waist circumference (cm), mean ± SD	100.9 ± 10.3
Mid-upper arm circumference (cm), mean ± SD	24.8 ± 2.2
Corrected arm muscle area (cm^2^), mean ± SD	39.4 ± 8.9
Calf circumference (cm), mean ± SD	36.7 ± 3.1
At risk of malnutrition,^f^*n* (%)	187 (18.8)
Loss of appetite,^g^*n* (%)	246 (24.6)
Serum albumin (g/L), mean ± SD	45.9 ± 2.7
Physical function	
Grip strength (kg), mean ± SD	31.1 ± 7.2
5 chair rises, mean ± SD	13.9 ± 4.5
Gait speed (m/s), mean ± SD	0.83 ± 0.2
Mobility limitations, *n* (%)	290 (29.2)
Activities of daily living problems, *n* (%)	221 (22.1)
Instrumental activities of daily living problems, *n* (%)	243 (24.3)
Frailty,^h^*n* (%)	236 (27.1)

FEV, forced expiratory volume; SD, standard deviation; BMI, body mass index.

a Classed as spending any time doing ≤3 of the following: (i) with family, friends, and neighbours, (ii) with friends/relatives on the telephone, (iii) in paid work, (iv) in voluntary work, (v) in a pub or club, (vi) attending religious services, (vii) playing cards, games or bingo, (viii) reading or (ix) attending class or (x) course of study.

b Self-report of feeling lonely; (i) strongly agree, (ii) agree, (iii) neither agree nor disagree, (iv) disagree or (v) strongly disagree.

c Self-report of being less active or much less active than an average man.

d Self-report of quality of sleep rated as: (i) excellent, (ii) good, (iii) fair, or (iv) poor.

e >20% of sites with 5.5-mm loss of attachment.

f Risk of malnutrition determined by Malnutrition Universal Screening Tool clinical parameters: low BMI, unintentional weight loss and acute disease.

g Simplified Nutritional Appetite Questionnaire score ≤15.

h Frailty based on at least 3 of 5 variables from the Fried frailty phenotype: (i) unintentional weight loss, (ii) exhaustion, (iii) low physical activity, (iv) weakness (grip strength) or (vi) slow walking speed.

## What has it found?

The BRHS has over 600 peer-reviewed publications and has extensively published on areas of cardiovascular health and mortality, including established and novel risk factors. Recent publications include conditions and morbidity related to CVD and older age, such as frailty,^31^ physical disability and periodontal disease.^29^ Since the previous update there has been additional emphasis on behavioural factors related to health and disease (e.g. sleep,^32^^,^^33^ diet^23^ and particularly physical activity^34^^,^^35^) which have been prospectively followed into the 40-year follow-up period. Moreover, being one of the very few population-based epidemiological studies of older adults to have a physical examination of oral health in 2010–12, the BRHS data have been used to provide estimates of the burden of poor oral health in older British men which, among other BRHS publications, have received considerable media attention.^36^ A full list of peer-reviewed publications is available at [https://www.ucl.ac.uk/british-regional-heart-study].

The newly collected 40-year follow-up data in the 2018 cohort demonstrates a high level of self-reported ill health and disease, including CVD, as the cohort has advanced into very old ages (78–99 years). Moreover, the number of individuals that have edentulism (no teeth) and wear dentures is similar to national data from England’s Adult Dental Health Survey,^28^ with an average two (±3 SD) teeth lost since 2010–12. In addition, comparison with the previous physical examination shows individual-level worsening of activities of daily living problems, risk of malnutrition and frailty status (14%, 17% and 20%, respectively) as the cohort has grown older. A summary of characteristics of the cohort sample in 2018 according to different domains of health, physical function, well-being and social factors, are presented in Table 3.

## What are the main strengths and limitations?

The strengths and limitations of the BRHS 40-year follow-up cohort remain broadly similar to those previously highlighted.^2^ A major strength of the BRHS is the collection of rich and detailed repeated measures. A key feature of this cohort is the follow-up since baseline (middle age) to the current 40-year follow-up into very old ages, allowing longitudinal investigations across the adult life course. To complement these longitudinal investigations, the measures have evolved with time to incorporate a wide range of conditions and determinants relevant to the health of ageing adults. However, due to the nature of this study, mortality is an inevitable determining factor of the reduced cohort sample size. Although this may be deemed as a limiting consequence of attrition, the strength remains as the enhanced phenotyping of very old men. Age and health status may be a factor that limited participants from attending the physical examination, but postal questionnaires were collected from those unable to be physically examined, which maintains the collection of detailed self-report data. The lack of female and ethnic minority representation limits the generalizability and comparability of the BRHS to White males. However, the British Women’s Heart and Health Study^36^ acts as a parallel female cohort, investigating the risk factors associated with CVD in women aged over 60 years. Similarly, the lack of recruitment from inner-city populations and towns with high mobility and from rural communities reduces the generalizability of the BRHS cohort to the whole UK population.

## Can I get hold of the data?

Study details and accessibility to questionnaires, data collection document, and relevant publications can be found on [https://www.ucl.ac.uk/british-regional-heart-study]. In addition, new collaborations continue to be welcomed and data sharing enquiries should be directed to Lucy Lennon [l.lennon@ucl.ac.uk].

## Ethics approval

Ethical approval for this study has been obtained from National Research Ethics Service (NRES) Committee London—Central, reference number: MREC/02/2/91. Participants provided informed written consent to the investigation, which was performed in accordance with the Declaration of Helsinki.

## Data Availability

See ‘Can I get hold of the data?’ above.
